# Macromolecular crowding amplifies allosteric regulation of T-cell protein tyrosine phosphatase

**DOI:** 10.1016/j.jbc.2022.102655

**Published:** 2022-10-31

**Authors:** May Thwe Tun, Shen Yang, Fabio Luis Forti, Eugenio Santelli, Nunzio Bottini

**Affiliations:** 1 Department of Biology, University of California San Diego, La Jolla, California, USA; 2 Department of Medicine, University of California San Diego, La Jolla, California, USA; 3 Department of Biochemistry, Institute of Chemistry, University of Sao Paulo, SP, Brazil

**Keywords:** allosteric regulation, protein phosphatase, autoimmunity, cancer, macromolecular crowding, tyrosine phosphatase, immuno-oncology, PTPN2, TC-PTP, regulation, CFP, cyan fluorescent protein, EGFR, epidermal growth factor receptor, IC, intracellular, IDR, intrinsically disordered C-terminal region, IFN-γ, interferon gamma, JAK–STAT, Janus kinase–signal transducer and activator of transcription, LLPS, liquid–liquid phase separation, mEGFP, monomeric enhanced GFP, NLS, nuclear localization signal, pNPP, *p*-nitrophenyl phosphate, PTK, protein tyrosine kinase, PTP, protein tyrosine phosphatase, TC-PTP, T-cell protein tyrosine phosphatase

## Abstract

T-cell protein tyrosine phosphatase (TC-PTP) is a negative regulator of T-cell receptor and oncogenic receptor tyrosine kinase signaling and implicated in cancer and autoimmune disease. TC-PTP activity is modulated by an intrinsically disordered C-terminal region (IDR) and suppressed in cells under basal conditions. *In vitro* structural studies have shown that the dynamic reorganization of IDR around the catalytic domain, driven by electrostatic interactions, can lead to TC-PTP activity inhibition; however, the process has not been studied in cells. Here, by assessing a mutant (^378^KRKRPR^383^ mutated into ^378^EAAAPE^383^, called TC45^E/A^) with impaired tail–PTP domain interaction, we obtained evidence that the downmodulation of TC-PTP enzymatic activity by the IDR occurs in cells. However, we found that the regulation of TC-PTP by the IDR is only recapitulated *in vitro* when crowding polymers that mimic the intracellular environment are present in kinetic assays using a physiological phosphopeptide. Our FRET-based assays *in vitro* and in cells confirmed that the effect of the mutant correlates with an impairment of the intramolecular inhibitory remodeling of TC-PTP by the IDR. This work presents an early example of the allosteric regulation of a protein tyrosine phosphatase being controlled by the cellular environment and provides a framework for future studies and targeting of TC-PTP function.

Protein tyrosine phosphatases (PTPs) regulate almost every major cellular process—ranging from cell cycle, growth, and differentiation to cellular metabolism by counterbalancing the action of protein tyrosine kinases (PTKs). Several PTPs are validated drug targets for multiple major diseases. However, the drugging of PTPs has lagged behind PTKs. Recent seminal reports have highlighted the potential promise of inhibitors that operate through noncompetitive mechanisms (1, 2, 3) and paved the way to several allosteric inhibitors of two medically relevant PTPs, SHP2 and PTP1B, progressing to clinical trials (4, 5). There is a renewed appreciation of the importance of understanding the biochemical modulation of PTP catalytic activity by their regulatory domains and other mechanisms (6, 7, 8, 9) in order to enhance the tractability of this family of enzymes.

T-cell protein tyrosine phosphatase (TC-PTP) is a ubiquitously expressed PTP belonging, along with PTP1B, to the NT1 subtype of nonreceptor PTPs, which has emerged as a target for oncology and autoimmunity (10, 11, 12). TC-PTP is a key regulator of immune signaling, inflammation, hematopoiesis, metabolism, and vesicle trafficking through dephosphorylation of SRC family kinases and control of Janus kinase–signal transducer and activator of transcription (JAK–STAT) signaling (13, 14). Loss-of-function variants of TC-PTP-encoding PTPN2 are major genetic determinants of autoimmunity in humans (15), and deletion of TC-PTP in T cells causes spontaneous autoimmunity (16). Deletion of TC-PTP enables spontaneous and implanted tumor rejection *via* a combined effect on T-cell signaling and interferon gamma (IFN-γ)–induced JAK–STAT signaling in the tumor (17). There is a strong interest in elucidating regulation of TC-PTP and inhibitor discovery (18). A competitive inhibitor of TC-PTP and PTP1B (Abbvie/Calico compound #182) has recently progressed to clinical trials in patients with advanced cancers (19).

Along with PTP1B, TC-PTP has an N-terminal catalytic domain immediately followed by a regulatory α-helix, denoted as α7, and a C-terminal ∼90 amino acid intrinsically disordered tail region (IDR) (20). Its most abundant 45 kDa form, often referred to as TC45, localizes to the nucleus owing to a dipartite localization signal near its C terminus, though it is known to shuttle between the nucleus and the cytoplasm in response to cellular stimuli (21, 22, 23). A negative regulatory role for the C-terminal tail under basal conditions has long been established (24, 25, 26, 27) and linked to the ability of TC-PTP to be activated upon localization to the appropriate sites (28, 29). Much of what is known about TC-PTP allostery at the structural level had until recently been inferred from its similarity to PTP1B. A pair of recent reports however have shown that, while the molecular mechanism by which α7 controls TC-PTP activity closely mirrors that seen in PTP1B (30), the mode of action of the IDR does not: based on mutagenesis, chemical crosslinking and nuclear magnetic resonance data, Singh *et al.* (31) proposed a model in which residues 300 to 387, C terminal to α7, wrap flexibly around the catalytic domain *via* intramolecular dynamic interactions, thus sterically restricting substrate access to the active site. A prominent role in facilitating the formation of this compact state is played by a stretch of basic residues ^377^RKRKRPR^383^ located near the protein’s C terminus that interact with a surface patch distal to the active site. Mutations or truncations that eliminate the positive charge in this region impair the inhibitory effect of the tail, as does increased ionic strength, confirming the essentially electrostatic nature of the interaction (25, 31).

The study of the properties of proteins in aqueous buffers has historically proved invaluable to the ultimate goal of understanding their behavior in the intracellular (IC) or extracellular milieu. In the last few decades, though, increasing attention has been drawn to the drawbacks of this approach (32, 33, 34). The inclusion of so-called crowders in the *in vitro* characterization of macromolecules has been invoked as a simple and easily accessible means to mimic the environment present in the relevant context (35); however, this approach has only been applied to SH2 domain–containing PTPs in two recent reports so far (36, 37). Here, we provide evidence that the use of polymeric crowders markedly shifts TC-PTP toward its autoinhibited form *in vitro* and is needed to recapitulate its behavior in the cell. The work presented here provides cellular validation of the recent intramolecular model of TC-PTP regulation, highlights the importance of taking into account the effects of the physicochemical properties of the medium in the *in vitro* study of PTPs, and offers new tools in the quest to better understand the physiological regulation of TC-PTP.

## Results

### TC-PTP exists in an autoinhibited state in cells, dependent on its C-terminal tail

**Figure 1:**
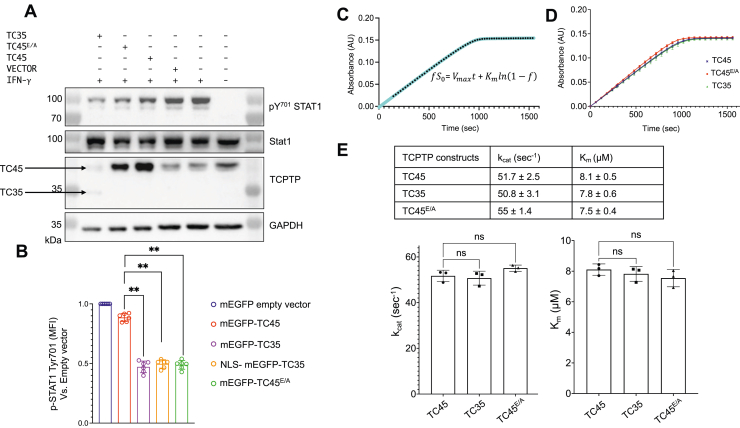
**Inhibition of TC-PTP activity is observed in cells but not in *in vitro* phosphatase assays.***A*, Western blot analysis of phospho-STAT1 in H358 cells overexpressing TC45, TC45^E/A^, and TC35 stimulated with or without IFN-γ for 1 h. The blots are representative of three independent experiments. *B*, quantification of mean fluorescence intensity of pY^701^-STAT1 from Phospho-STAT1 flow cytometry assays. Cells are transfected with mEGFP-TC45 vectors. *C*, representative calculation of kinetic parameters from absorbance *versus* time data. *Cyan*: fitted curve; •: experimental data; data shown are in 60 s intervals. *D*, graph depicts kinetic curves of TC45 (*blue*), TC45^E/A^ (*red*), and TC35 (*green*). Data are from three independent replicates. *E*, *top*, summary of kinetic parameters determined as in *C*. Data are the mean of three independent measurements. Mean ± SD. *Bottom*, bar graphs showing *K*_*m*_ and *k*_cat_ as mean ± SD for TC45, TC45^E/A^, and TC35 (ordinary ANOVA; ns). IFN-γ, interferon gamma; mEGFP, monomeric enhanced GFP; ns, not significant; STAT, signal transducer and activator of transcription; TC-PTP, T-cell protein tyrosine phosphatase.

Previous studies have shown that a 33 kDa N-terminal fragment of TC-PTP encompassing only the catalytic domain is more active than the full-length enzyme, and that truncation of ∼20 C-terminal residues, comprising the ^377^RKRKRPR^383^ motif, is sufficient to restore full activity (25, 31). However, the relevance of this mechanism of regulation to cancer-relevant JAK–STAT signaling has not been established. Western blots with anti-pY^701^STAT1 antibodies and Phospho-STAT1 flow cytometry show that suppression of TC-PTP activity with compound #182 (19) after IFN-γ stimulation in NCI-H358 lung cancer cells led to increased STAT1 Y^701^ phosphorylation (Fig. S1). To directly demonstrate that TC-PTP is autoinhibited in cells under basal conditions, we then determined the IFN-γ stimulation–induced phosphorylation state of STAT1 Y^701^ after overexpression of TC-PTP in NCI-H358 cells. Only a modest decrease in phosphorylation level was seen when overexpressing wildtype full-length TC-PTP (Fig. 1*A*, *third lane from left*, as TC45). In contrast, overexpression of TC-PTP 1 to 304 (TC35) or a mutant in which residues ^378^KRKRPR^383^ are mutated to EAAAPE (TC45^E/A^) led to markedly higher dephosphorylation of STAT1 Y^701^, despite lower TC-PTP overexpression levels. This result shows that TC35 and TC45^E/A^ are both gain-of-function mutants, in agreement with the finding that TC-PTP is inhibited by its C-terminal tail as described previously. We confirmed this result by pY^701^STAT1 flow cytometry using the same three variants N-terminally fused to the nondimerizing form of monomeric enhanced GFP (mEGFP). In addition, since TC35 lacks a nuclear localization signal (NLS), we generated a TC35 construct carrying the NLS from the SV40 large T-antigen N terminal to the mEGFP fusion, allowing a more direct comparison between the different forms of the enzyme. pY^701^STAT1 flow cytometry confirmed that overexpression of TC35 and TC45^E/A^, but not TC45, decreases phosphorylation of STAT1 (Fig. 1*B*), regardless of the presence of the NLS.

### The C-terminal tail has no effect on TC-PTP activity *in vitro* in aqueous buffers

In order to better understand the function of the tail, we sought to study the behavior of TC-PTP and its mutants *in vitro*. We performed phosphatase assays using the epidermal growth factor receptor (EGFR)–derived 12-mer peptide DADE-pY-LIPQQG and purified bacterially expressed TC-PTP. The dephosphorylation reaction can be followed by monitoring the conversion of phosphotyrosine to tyrosine, taking advantage of the higher absorbance of the latter around 282 nm (38). We calculated *V*_max_ and *K*_*m*_ by fitting the data to the implicit form of the integrated Michaelis–Menten equation *fS*_*0*_*= V*_max_ *t + K*_*m*_ ln*(1-f)*, where *S*_*0*_ is the initial substrate concentration and *f* is the fraction of substrate converted at the time *t*. Surprisingly, we observed no significant differences in activity between TC45, TC35, and TC45^E/A^ (Fig. 1, *C*–*E*). Similar results were obtained using *p*-nitrophenyl phosphate (pNPP) as substrate in classical Michaelis–Menten kinetic assays, as shown in Fig. S2.

### The C-terminal tail has an inhibitory effect on TC-PTP activity *in vitro* in the presence of macromolecular crowding agents

**Figure 2:**
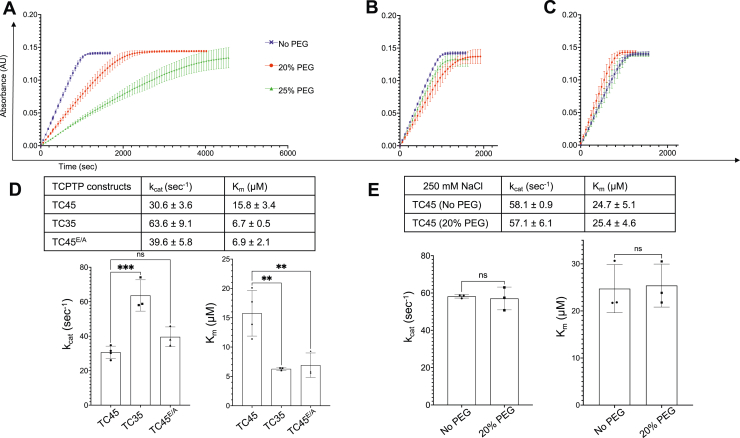
**PEG 3350 in the reaction buffer affects TC45 but not TC45**^**E/A**^**and TC35 kinetics.***A*, graph depicts the kinetic curves of TC45 with 0 to 25% PEG 3350; data are from three independent replicates for each concentration of PEG 3350. Color scheme is as for Figure 1*D*. *B*, TC45^E/A^ with 0 to 25% PEG 3350. *C*, TC35 with 0 to 25% PEG 3350. *D*, *top*, summary of kinetic parameters for 20% PEG 3350 determined as for Figure 1*C*. *Bottom*, bar graphs depicting *K*_*m*_ and *k*_cat_ mean ± SD for TC45, TC45^E/A^, and TC35 at 20% PEG 3350. *E*, *top*, summary of kinetic parameters of TC45 with and without 20% PEG 3350 at 250 mM NaCl. *Bottom*, bar graphs depicting *K*_*m*_ and *k*_cat_ mean ± SD (ordinary ANOVA; ∗∗<0.02; ∗∗∗*p* < 0.001). ns, not significant.

To understand the apparent inconsistency between the cellular and *in vitro* data, we turned to investigating the potential effect of molecular crowding agents on the enzymatic activity of the different forms of TC-PTP. When we repeated the kinetic assays with the 12-mer peptide in the presence of 20 or 25% PEG 3350, we saw a dose-dependent inhibition of the activity of TC45 but not TC35, whereas TC45^E/A^ showed slight inhibition (Fig. 2, *A*–*C*), in line with the observations in cells. The reduction in TC45 activity at 20% PEG results from both a 1.7-fold decrease in *k*_cat_ and a twofold increase in *K*_*m*_ (Fig. 2*D*). To verify that PEG does not irreversibly inactivate TC45, we incubated TC45 at 1 μM in the same 20% PEG 3350 conditions used for the kinetic assay before diluting it to 4 nM to perform the assay in the absence of PEG. TC45 showed full recovery of activity compared with samples that were not incubated with PEG (Fig. S3). As the effect of the tail is dependent on electrostatic interactions with the catalytic domain (31) and it drastically drops with increasing ionic strength (25), we carried out the same assays in a buffer containing 20% PEG while increasing the NaCl concentration to 250 mM. As expected, along with the increased *K*_*m*_ commonly associated with higher ionic strength in PTPs, there was little or no inhibitory effect from the presence of PEG (Fig. 2*E*). Therefore, the presence of a molecular crowding agent that mimics the cellular environment is necessary to recapitulate *in vitro* the cellular behavior of full-length wildtype TC-PTP. PEG is generally regarded as inert; however, the possibility of dynamic weak interactions between protein and other solutes when using various crowding agents has to be taken into consideration (35, 39, 40). As the methylene groups of PEG can expose the protein to a hydrophobic local environment, we tested the effect of a more hydrophilic and chemically unrelated polymer in our kinetic assay. Fig. S4 shows a similar effect of 5% Ficoll on the kinetic curves for TC45 and TC45^E/A^, strongly suggesting that the excluded volume effect because of steric repulsions is at least in part responsible for the inhibition of TC-PTP activity by its IDR. Finally, we sought to confirm these findings by performing the phosphatase assays in the same buffers with a phosphopeptide derived from the activation loop of lymphocyte-specific PTK (Lck), EDNEpYTAREGA, as substrate. Overall, as shown in Fig. S5, the kinetic parameters, in the aqueous buffer show, similar *k*_cat_ and more than doubled *K*_*m*_ values compared with those obtained with the EGFR-derived phosphopeptide. The presence of PEG leads to a decrease in *K*_*m*_ for TC45^E/A^ and especially TC35, likely because of the excluded solvent effect, and an increase for TC45, whereas the differences in *k*_cat_ are less significant but still in broad agreement with the pattern observed for the EGFR peptide. These results therefore confirm the PEG-dependent inhibitory role of the TC-PTP tail described previously.

### PEG 3350 as a cosolute favors a more compact state for TC45

**Figure 3:**
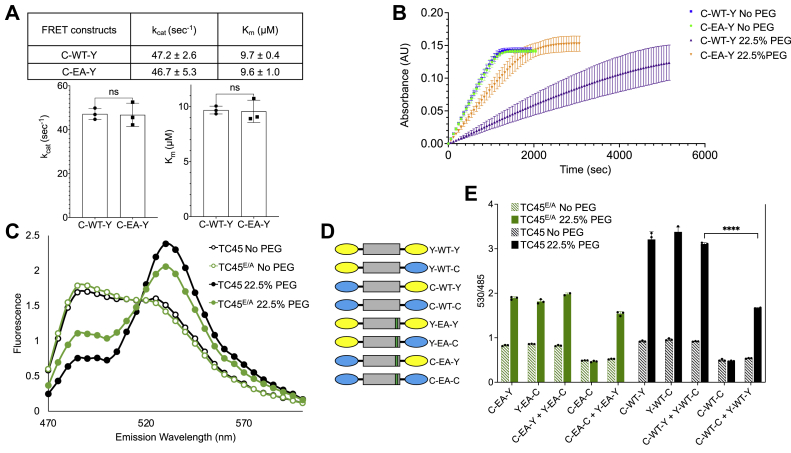
**PEG 3350 promotes inhibition of TC-PTP activity by its C-terminal tail by a combination of intramolecular and intermolecular interactions.***A*, *top*, summary of kinetic parameters determined as for Figure 1*C*. *Bottom*, bar graphs depicting *K*_*m*_ and *k*_cat_ mean ± SD for CFP-TC45-YFP and CFP-TC45^E/A^-YFP (ordinary ANOVA; ns). *B*, kinetic curves for CFP-TC45-YFP (*blue*) and CFP-TC45^E/A^-YFP (*green*) at 0% PEG 3350 and CFP-TC45-YFP (*purple*) and CFP-TC45^E/A^-YFP (*orange*) at 22.5% PEG 3350. Mean ± standard deviation of three independent experiments is shown. *C*, a representative fluorescence emission of CFP-TC45-YFP (*black*) and CFP-TC45^E/A^-YFP (*green*) in a kinetic buffer with no PEG (*empty circles*) or 22.5% PEG 3350 (*solid circles*). *D*, schematic representation of the fusion proteins used in *E*. *E*, comparison of the ratio R of the emission at 530 nm (YFP)/485 nm (CFP) for the TC45 and TC45^E/A^ fluorophore fusion samples in the absence or presence of PEG 3350. Representative fluorescence emission spectra used for the calculation of R are shown in Fig. S5 (ordinary ANOVA; ∗∗∗∗*p* < 0.0001). CFP, cyan fluorescent protein; ns, not significant; TC-PTP, T-cell protein tyrosine phosphatase.

The effect of the tail on TC-PTP catalytic activity is linked to a reorganization of the protein into a compact state in which the N and C termini are brought closer together, albeit not in close contact (31). In order to investigate the connection between kinetic data and molecular configuration, we implemented an FRET-based approach by generating constructs for bacterial expression in which the amino and carboxy termini of TC45 and TC45^E/A^ are fused to cyan fluorescent protein (CFP) or YFP *via* a short linker. We denote the corresponding isolated proteins as C-WT-Y for CFP-TC45-YFP and C-EA-Y for CFP-TC45^E/A^-YFP. When CFP is excited around 430 nm, the expected CFP emission in the absence of FRET is at 485 nm; however, the more the two ends of TC-PTP are brought together because of the compact state of the autoinhibited form, the more fluorescence emission will occur around 530 nm (41). We first sought to confirm that the fusions do not appreciably affect the activity of TC45 or the effect of PEG on it. Similar to the proteins without fluorophores, there was no difference between TC45 and TC45^E/A^ (Fig. 3*A*) in kinetic buffer, whereas Figure 3*B* shows that a difference between the activities of C-WT-Y and C-EA-Y adequate for further experiments was observed at 22.5% PEG 3350. FRET assays in the absence of PEG showed a low-emission signal at 530 nm, consistent with a stochastic conformation of the intrinsically disordered tail (Fig. 3*C*). Interestingly, we observed no significant difference in fluorescence emission between TC45 and TC45^E/A^, in agreement with the *in vitro* kinetics data. In the presence of PEG, there was a significant shift toward 530 nm for C-WT-Y and, to a lesser extent, C-EA-Y, mirroring the slower kinetics shown in Figure 3*B*. These data are consistent with a link between crowding, catalytic activity, and restructuring of the C-terminal tail around the PTP domain.

### PEG 3350 as a cosolute promotes TC-PTP clustering

A common effect of crowding agents is to promote the clustering of macromolecules because of the excluded volume effect (35, 42), which can directly influence their catalytic activity by limiting substrate access to the active site. To better understand the mechanism by which crowding acts on TC-PTP, we generated C- and N-terminal CFP and YFP fusions in all possible combinations for both TC45 and TC45^E/A^, including C-C and Y-Y species to be used as controls (Fig. 3*D*). Figure 3*E* shows a schematic view of the ratio (R) between the fluorescence emission at 530 and 485 nm for various individual fusions or their equimolar mixtures, while keeping the total protein concentration at 10 nM. In kinetic buffer with no PEG, all R values for corresponding TC45 and TC45^E/A^ pairs were similar, as noted previously for C-WT-Y and C-EA-Y, whereas all C-Y and Y-C species showed a small increase in emission at 530 nm when compared with C-C fusions, presumably because of the fluorophores at opposite ends of the protein being close enough to generate a FRET signal even in the absence of a compact and autoinhibited structure. Considering first TC45^E/A^ (*green bars*), the presence of PEG led to a sharp increase in R for all species or mixtures that were able to generate a FRET signal. Since there cannot be intramolecular FRET in the C-EA-C + Y-EA-Y sample, we attribute this to intermolecular association induced by the crowding effect of PEG, with the caveat that we could not independently measure the effect of the medium on FRET efficiency or on CFP and YFP interaction. As the increase in R and emission at 530 nm (Fig. S6) was similar for all TC45^E/A^ fusions (and under the assumption that any intermolecular FRET depends only on the density of fluorophores in the “associated” state), PEG did not appear to promote the transition to the autoinhibited form. A different picture appeared for TC45: while the value of R was similarly elevated for the C-C + Y-Y fusion sample when compared with TC45^E/A^, a much larger jump was observed for C-WT-Y, Y-WT-C, and their equimolar combination, which can be explained by a contribution to FRET from the intramolecular proximity between N and C termini, as predicted by the available structural model.

### Both TC-PTP clustering and intramolecular IDR reorientation occur in cells

**Figure 4:**
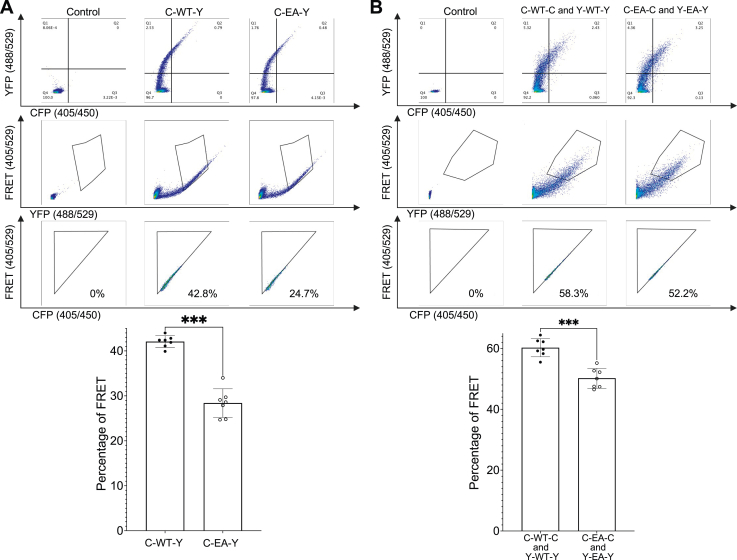
**FRET measurements of TC45 and TC45**^**E/A**^**in cells display both intramolecular and intermolecular interaction.***A*, *left*, representative FACS plots for control, CFP-linker-YFP, CFP-TC45-YFP, and CFP-TC45^E/A^-YFP. *Top*, double positive cells for CFP and YFP expression. *Middle*, selection of FRET-positive cells excluding the false-positive signal because of direct YFP excitation. *Bottom panel*, triangular gating containing FRET-positive cells. *Right*, bar graphs summarizing the results from the panels on the *left*. Representative examples of the FACS plots used to determine the gating strategy for these measurements are shown in Fig. S6*A*. *B*, *left*, three panels are representative FACS plots for the overexpression of NLS-CFP and ^NLS^-YFP, CFP-TC45-CFP and YFP-TC45-YFP, CFP-TC45^E/A^-CFP and YFP-TC45^E/A^-YFP. *Top*, *middle*, and *bottom panels* are as described above. *Right*, bar graphs summarizing the results from the panels on the *left*. Representative examples of the FACS plots used to determine the gating strategy for these measurements are shown in Fig. S6*B*. Data represent seven replicates and were analyzed with Kruskal–Wallis test (∗*p* < 0.05; ∗∗*p* < 0.01). CFP, cyan fluorescent protein; FACS, fluorescence-activated cell sorting; NLS, nuclear localization signal.

Next, we investigated the effect of the cellular environment on the association and conformation of TC-PTP by FRET-based flow cytometry. Plasmids carrying CFP-TC45-YFP and CFP-TC45^E/A^-YFP fusions were transfected into NCI-H358 cells. CFP or YFP alone and CFP fused to YFP *via* a short linker (CFP-linker-YFP) served as controls. As demonstrated in Figures 4 and S7, intramolecular FRET was detected in cells overexpressing CFP-TC45-YFP, whereas cells overexpressing CFP-TC45^E/A^-YFP showed reduced FRET signal (Fig. 4*A*). The results align with the *in vitro* FRET results described previously and, together with the evidence of a gain-of-function phenotype for TC45^E/A^ (Fig. 1*A*), support a link between the IDR–PTP domain interaction and inhibition of TC-PTP activity in cells. In addition, we cotransfected CFP-TC45-CFP and YFP-TC45-YFP (CFP-TC45^E/A^-CFP and YFP-TC45^E/A^-YFP) to probe any intermolecular interaction occurring inside the cells. Similar to the intramolecular interaction results, the intermolecular interaction of TC45^E/A^ displayed a mild but significant decrease compared with TC45 (Fig. 4*B*). Overall, these results are in good agreement with those from the *in vitro* FRET in the presence of PEG and show that the inclusion of a crowding agent is necessary to fully recapitulate the cellular behavior of TC-PTP *in vitro*.

## Discussion

**Figure 5:**
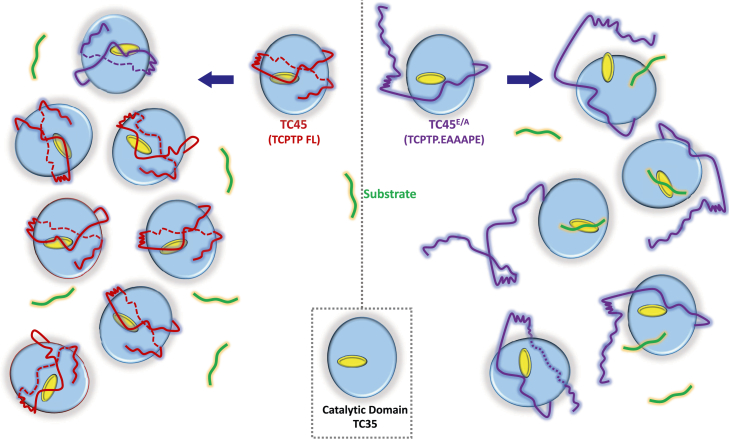
**Schematic representation of the effect of crowding or the cellular environment on the structure and activity of TC-PTP**. *Light blue*, catalytic domain. *Yellow*, active site. *Red and purple*, IDR, respectively, for TC45 and TC45^E/A^. *Green*, substrate. IDR, intrinsically disordered C-terminal region; TC-PTP, T-cell protein tyrosine phosphatase.

TC-PTP is a recognized clinically relevant signaling enzyme, yet there is a scarcity of data in the scientific literature from *in vitro* studies of its properties. In this work, we provide evidence that allosteric regulation of TC-PTP catalytic activity by its C-terminal IDR *in vitro* requires mimicking of the cellular environment with macromolecular crowders, an observation that should inform future mechanistic studies and might explain why this enzyme remains so far underinvestigated. Figure 5 summarizes our current interpretation of the experimental data.

Earlier work had established that the IDR can modulate the phosphatase activity in TC-PTP even in dilute solutions in aqueous buffers. Zander *et al.* (24) observed a complex substrate-specific effect, whereas Hao *et al.* (25) suggested a dependence on the substrate’s size and noted that the effect is maximal at very low and largely subsides at near physiological ionic strengths. Mattila *et al.* (27) were able to restore full enzymatic activity using positively charged competitors of the regulatory interaction using DiFMUP as substrate. Finally, Singh *et al.* (31) measured a threefold increase in *k*_cat_, though accompanied by a twofold increase in *K*_*m*_, with the same EGFR-derived phosphopeptide substrate used in this work when they truncate the protein to include the PTP domain and α7 only. Given the sensitivity of TC-PTP to its environment, it is perhaps not surprising that different groups, utilizing different assays, protein isolation procedures, kinetic conditions, substrates, additives (possibly including the substrates themselves), and materials, come to seemingly contradictory conclusions. Clearly, the field could benefit from a systematic assessment of the factors that influence the behavior of TC-PTP in dilute solutions. Here, focusing on the influence of fairly high concentrations of polymers on the allosteric regulation of the enzyme, we come to the unambiguous conclusion that, at least in the conditions used in this work, synthetic mimics of the cellular environment are able to reproduce the protein’s cellular behavior and propose that the crowded surroundings in the cell are at least in part responsible for it. While the roughly twofold inhibition we observe in PEG buffers may appear small, its extent is in line with, for example, the effect of the allosteric α7 helix in the context of the isolated PTP domain in TC-PTP (30) or PTP1B (43) and is not unusual in *in vitro* studies of PTP allostery (9, 44). Moreover, we cannot predict the size of the effect in cells or its consequences on signaling pathways simply from *in vitro* data. We believe, however, that the connection between the *in vitro* and cellular data presented in this work can be a starting point to stimulate a more quantitative approach to the investigation of TC-PTP regulation in cells or *in vivo*.

Mechanistically, the stabilization of a compact state in a crowded buffer can be explained on the basis of entropic factors alone (45). However, as the IDR–PTP domain interaction involves neutralization of charges on the surface of both domains, one possibility that can be explored is that a more hydrophobic environment *via* dynamic interactions with cosolutes such as other proteins in the cell, PEG in the buffer, or even other TC-PTP molecules as a result of crowding, would stabilize the autoinhibited structure. In turn, the compact state will have more or less propensity to self-associate, thus giving rise to a complex interplay between different contributing factors. Particularly worthy of further investigation is whether the formation of macromolecular clusters can be the basis for substrate selectivity.

In summary, we have obtained evidence in support of the notion that the reconfiguration of the IDR of TC-PTP around its catalytic domain to form a sterically autoinhibited structure is dependent on the presence of crowded surroundings such as the ones found in the cell. This may have implications for the study of other similar systems. The investigation of protein folding and stability in crowded media is now a well-established field of research (40, 45, 46). While a review of the literature on the less explored effects of crowding on IDP/IDRs has nonetheless revealed a complex set of disparate behaviors (47), the issue remains so far unaddressed in the field of PTPs and generally understudied for other enzyme classes. Two recent articles have brought to light the role of liquid–liquid phase separation (LLPS) on the function of the close homologs SHP2 and SHP1, with profound implications for their study and the development of strategies for their therapeutic manipulation (36, 37). We were unable to detect LLPS in cells using overexpressed mGFP fused to TC-PTP (data not shown), suggesting the presence of a looser association between molecules or phase-separated droplets too small to be visualized. Thus, we present here a workflow and the molecular tools for the analysis of TC-PTP allosteric regulation and its exploitation for drug screening *in vitro* and in cells that does not rely on the assumption of the presence of LLPS. We hope this work will be valuable for future research toward the elucidation of TC-PTP function and that this approach will be applicable to other systems.

## Experimental procedures

### Antibodies and reagents

Rabbit monoclonal antibodies against Phospho-STAT1 (Tyr701), Stat1, GAPDH, and Phospho-STAT1 (Tyr701) antibody (phycoerythrin conjugate) were purchased from Cell Signaling Technology. Rabbit monoclonal antibody against TC-PTP (ab129070) was purchased from Abcam. Polyethylenimine was ordered from Sigma. EGF receptor peptide (DADE-pY-LIPQQG) was purchased from GenScript. Cell Line Nucleofector Kit T was from Lonza. eBioscience Fixable viability Dye eFluor 780 (Invitrogen) and IC fixation buffer were purchased from Thermo Fisher Scientific.

### Cell culture, treatment, and transfection

NCI-H358 cells were obtained from American Type Culture Collection and maintained in RPMI1640 (Corning) supplemented with 10% fetal bovine serum and 1% penicillin–streptomycin at 37 °C in 5% CO_2_ atmosphere. For inhibitor treatment, the cells were pretreated with 30 μM compound #182 for 30 min and then stimulated with or without 1000 U/ml hIFN-γ for the indicated time. For transfection of NCI-H358 cells with pCG-TC-PTP vectors, five million cells were transfected with 3 μg of DNA using Cell Line Nucleofector Kit T (Lonza), program X-001. At 24 h, the cells were starved for another 12 h and then stimulated with or without IFN-γ for the indicated time.

### Plasmid construction

A pEF FRET vector containing N-terminal CFP and C-terminal-YFP connected by a (GGGGS)_3_ linker, pEF EGFP vector, pEF mEGFP-TC-PTP wildtype plasmids carrying mEGFP 5′ or 3′ to the TC-PTP coding sequences was ordered from GenScript. pEF-mEGFP (EGFP containing a A206K mutation) vector was constructed by site-directed mutagenesis by using pEF-EGFP vector as a template, and its mutant plasmids were generated by standard site-directed mutagenesis. pCG-TC45 and empty pCG vectors were a gift from Prof Tony Tiganis. An ORF encoding for full-length TC45 was amplified and cloned into pET28a as a thrombin cleavable N-terminal six-histidine fusion using the NdeI and XhoI sites to generate pET28-TC45. TC35 and TC45^E/A^ mutants were generated by standard site-directed mutagenesis techniques. SV40 large T-antigen NLS (DPKKKRKV) coding sequence was added into pEF-mEGFP-TC35 plasmid 5′-terminal to mEGFP to get the pEF-NLS-mEGFP-TC35 by standard site-directed mutagenesis. pET28-mEGFP-TC45 plasmids were generated using the 3′ terminal EcoRI and 5′ terminal KpnI sites after engineering a KpnI site to replace the NdeI site into pET28a. These plasmids were used for preliminary assessments of the effect of adding the fused proteins on the catalytic activity of TC45 (not shown). N-terminal CFP and YFP were PCR amplified and introduced into this construct using the KpnI and a BamHI site 3′ to the mEGFP coding region to generate pET28-CFP-TC-PTP and pET28-YFP-TC-PTP. C-terminal CFP and YFP were PCR amplified from pEF FRET and introduced into pET28-TC-PTP-mEGFP *via* the BamHI and an engineered AgeI site overlapping the last codon of the TC-PTP coding sequence. To introduce a C-terminal CFP/YFP into the N-terminal CFP/YFP fusion constructs, we directly cloned them using the EcoRI site 3′ of the stop codon and a ScaI site internal to the TC-PTP coding region. The same fusions were transferred into pEF vector using the equivalent KpnI and EcoRI sites in both vectors. All clones were confirmed by sequencing.

### Protein expression and purification

Bacterial cultures carrying the expression plasmids were grown in LB broth in the presence of 50 μg/l kanamycin at 37 °C to an absorbance of 0.6 at 600 nm, and protein expression was induced with 0.25 mM IPTG at 18 °C for 18 h. The cells were harvested by centrifugation and lysed in 20 mM Tris–HCl (pH 8.0), 300 mM NaCl, 200 μg/ml lysozyme, and protease cocktail inhibitor (Pierce). After digestion with DNaseI, the lysate was centrifuged at 14,500 rpm for 1 h, and the soluble fraction of the cell extract was purified by nickel–nitrilotriacetic acid affinity chromatography. The eluted fraction was further purified by anion (TC35 and TC45E/A) or cation (TC45) exchange chromatography on Q or S HP column (Cytiva), respectively, on an NGC system (Bio-Rad). The relevant fractions were digested with thrombin overnight on ice, the reaction was stopped by running the protein through a Benzamidine Sepharose (Cytiva) column, and the protein was concentrated followed by a final cleanup step by size-exclusion chromatography. The purity was assessed to be >95% by SDS-PAGE. The proteins were concentrated in 20 mM Tris–HCl (pH 8.0), 500 mM NaCl, and stored at −80 °C.

### Phosphatase activity assays

The EGFR- and Lck-derived phosphopeptides (DADEpYLIPQQG and EDNEpYTAREGA) were purchased from GenScript and Biomatik, respectively. For phosphatase assays using the EGFR peptide, the protein was incubated for 1 h (2 h for recovery after Ficoll incubation, see later) at 4 nM concentration at 30 °C in kinetic buffer (50 mM Tris–HCl [pH 7.0], 125 mM NaCl, 0.01% Tween-20, and 1 mM DTT) with or without 20 to 25% PEG 3350 (Anatrace) or 5% Ficoll-400 (EMD), and the reaction was initiated by mixing 196 μl of protein with 4 μl of 10 mM EGFR peptide pre-equilibrated at 30 °C. The dephosphorylation reaction was monitored for 6000 s or until a stable plateau was reached by continuously recording the difference in absorbance between 282 and 294 nm with a Thermo Scientific GENESYS 150 UV–visible spectrophotometer at 30 °C. Kinetic parameters were calculated according to the equation in Figure 1 using GraphPad Prism 9.0 (GraphPad Software, Inc). In practice, absorbance data were directly fit to the implicit equation y = (A-E) ∗ x + B ∗ ln(1-C ∗ (y-D + E ∗ x)) + D, where x and y are time and absorbance, respectively, A and B are *V*_max_ and *K*_*m*_ in absorbance units, C, D, and E are parameters that are allowed to be fit to account for small experimental deviations from their ideal values of C = 1/substrate concentration, D = 0 (absorbance at *t* = 0) and E = 0 (absorbance drift over time at completion of the reaction). Absorbance values were then converted to concentrations using the experimentally determined value ε = 0.747 AU mM^−1^ cm^−1^. For TC45 activity recovery after incubation in PEG or Ficoll-400, TC45 was incubated at 1 μM in a kinetic buffer containing 20% PEG 3350 or 5% Ficoll-400 for 1 h at 30 °C prior to dilution to 4 nM in the kinetic buffer as described previously.

pNPP dephosphorylation assays were performed in reaction buffer (100 mM Tris–HCl [pH 7.0], 50 mM NaCl, 0.01% Tween-20, and 1 mM DTT) with or without 20% PEG 3350. The protein was diluted to 4 nM in the reaction buffer and incubated at 30 °C for 1 h prior to reaction. Various concentrations of pNPP (0, 1, 2.5, 5, 10, 25, 50, and 100 mM) were prepared in deionized water from a stock buffered at pH 7.0. About 90 μl of protein and 10 μl of pNPP were mixed in a 96-well clear assay microplate at 0, 10, 20, 30, 40, 50, and 60 min at 30 °C, and the reaction was stopped at 60 min by adding 100 μl of 1 M NaOH followed by measuring the *p*-nitrophenol absorbance at 405 nm. Initial reaction rates were then calculated by linear regression, and the kinetic parameters (*K*_*m*_ and *V*_max_) were calculated in GraphPad Prism 9.0. Absorbance values were converted to concentrations using the experimentally determined value of 4.7 AU⋅mM^−1^.

### Western blotting

NCI-H358 cells were washed twice with cold PBS and lysed in radioimmunoprecipitation buffer (Thermo Fisher), supplemented with proteinase inhibitor and 1 mM PMSF, and incubated on ice for 15 min. The lysate was centrifuged for 10 min at 4 °C at 15,000 rpm. The supernatant was collected and boiled in a Laemmli sample buffer (Bio-Rad) containing 50 mM β-mercaptoethanol. Finally, the proteins were separated by SDS-PAGE and then transferred to nitrocellulose membrane. Target proteins were probed with specific antibodies and visualized by chemiluminescence.

### *In vitro* FRET assay

For FRET assays, the proteins were diluted to 10 nM in kinetic buffer, with or without 22.5% PEG 3350, in 96-well solid black polystyrene microplates (Corning) and incubated for 1 h at 30 °C. The fluorescence emission was measured from 470 to 600 nm with a SpectraMax M2^e^ plate reader (Molecular Devices) with an excitation wavelength of 400 nm. All samples were prepared in triplicate.

### Flow cytometry

FRET signaling was analyzed by flow cytometry as described previously (Fig. S7 and (48)). Briefly, NCI-H358 cells were transfected with NLS-CFP, NLS-YFP, CFP-TC45-YFP, and CFP-TC45^E/A^-YFP constructs in a 6-well plate with polyethylenimine. The signal from cells transfected with the CFP-linker-YFP construct was used as a basis to detect the intramolecular FRET signal. The cells cotransfected with CFP-TC45-CFP and YFP-TC45-YFP, or CFP-TC45^E/A^-CFP and YFP-TC45^E/A^-YFP constructs were used for intermolecular FRET signal analysis. NLS-CFP and NLS-YFP cotransfected cells served as a basis to detect the intermolecular FRET signal. Cells were collected after 24 h and washed with a fluorescence-activated cell sorting buffer (2.5% fetal bovine serum and 1 mM EDTA in PBS). The cells were stained with Fixable Viability Dye eFluor 780 for 20 min at room temperature, washed, and suspended for FRET flow analysis using a Bio-Rad ZE5 flow cytometer.

For Phospho-STAT1 flow cytometry, five million NCI-H358 cells were nucleofected with 5 μg of mEGFP-TC-PTP and mutants by using Cell Line Nucleofector Kit T. After 24 h of transfection, cells were starved for 12 h and stimulated with or without 1000 U IFN-γ. Cells were harvested and washed with a FACS buffer. After staining with Fixable Viability Dye eFluor 780 for 20 min at room temperature, the cells were fixed with an IC fixation buffer at room temperature for 15 min and with cold 100% methanol at 4 °C for 15 min. Permeabilized cells were washed with Perm/Wash buffer, blocked with FC blocker (BD Pharmingen) for 15 min at room temperature, and stained with pY^701^-STAT1 Rabbit antibody (phycoerythrin conjugate) for 1 h at room temperature. After washing, cells were suspended and analyzed by Bio-Rad ZE5 flow cytometry.

### Statistical analyses

Statistical analyses were performed in GraphPad Prism 9.0.2. Ordinary one-way ANOVA was used to assess significance for variables that were found to be normally distributed based on Shapiro–Wilk test. For the nonparametric statistics, the Kruskal–Wallis test was used.

## Data availability

All data supporting the findings of this study are found within the article and its supporting information. The original data related to the findings of this study are available from the corresponding author upon reasonable request.

## Supporting information

This article contains supporting information.

## Conflict of interest

N. B. has equity interests in Nerio Therapeutics, a company that may potentially benefit from the research results, and receives income from the company for consulting. The terms of this arrangement have been reviewed and approved by the University of California, San Diego in accordance with its conflict of interest policies. All other authors declare that they have no conflicts of interest with the contents of this article.
