# Supplementary material for: Macromolecular crowding amplifies allosteric regulation of T-cell protein tyrosine phosphatase
Source: J Biol Chem. 2022 Oct 31;298(12):102655. doi: 10.1016/j.jbc.2022.102655 (PMC9720572; doi:10.1016/j.jbc.2022.102655)
Supplement: Supporting information [file mmc1.docx]

**Macromolecular crowding amplifies allosteric regulation of T-Cell Protein Tyrosine Phosphatase**

May Thwe Tun, Shen Yang, Fabio Luis Forti, Eugenio Santelli, Nunzio Bottini

Supporting Information

Supplemental Figures:

Figure S1

Figure S2

Figure S3

Figure S4

Figure S5

Figure S6

Figure S7

**S1**: (A) Top: Western blot analysis of phospho-STAT1 in NCI-H358 cells that were pretreated with Compound #182 before stimulating them with IFN-γ for one hour. Molecular weight marker values are indicated on the right. The blot is representative of three independent experiments. Right: Quantification of the western blots shown on the left (Ordinary ANOVA; *: < 0.03). (B) Left: Analysis of Phospho-STAT1 flow cytometry for NCI-H358 cells that were pretreated with Compound #182 before stimulating them with IFN-γ for one hour. Right: Quantification of flow cytometry shown on the left. (Mann-Whitney; **: < 0.002).

**S2:** Top: summary of kinetic parameters of TC45 and TC45^E/A^ with pNPP as substrate. Values are from five independent experiments. Middle: Michaelis–Menten curves. Each point is the average of five measurements, bars represent SD. Bottom: bar graphs depicting K_m_ and k_cat_ mean ± SD for TC45 andTC45^E/A^. (Mann-Whitney Test; ns= not significant)

**S3**: Top: summary of kinetic parameters of TC45 in no PEG, 20% PEG 3350 buffer, and activity recovery assay after incubation in 20% PEG 3350 using the EGFR peptide as substrate. Parameters were calculated as in Figure 1C. Bottom: bar graphs depicting K_m_ and k_cat_ mean ± SD for the three experiments mentioned above. (Ordinary ANOVA; *, <0.03; ***, p < 0.0002; ns= not significant).

**S4**: Top: summary of Michaelis-Menten parameters in kinetic buffer containing 5% Ficoll-400 using the EGFR peptide as substrate, determined as in Figure 1C. Bottom: bar graphs depicting K_m_ and k_cat_ mean ± SD for TC45, TC45^E/A^, and TC35 at 5% Ficoll-400 (Ordinary ANOVA; *, <0.03, **, < 0.02; ****, p < 0.0001).

**S5: Kinetic analysis of TC-PTP and its mutants with the Lck-derived phosphopeptide substrate in the presence or absence of 20% PEG3350.** (A) Graph depicts the kinetic curves of TC45 with 0 (blue) or 20% (red) PEG-3350; Data are from three independent replicates for each mutant and concentration of PEG-3350. (B) TC35 with 0 or 20% PEG-3350. (C) TC45^E/A^ with 0 or 20% PEG-3350. (D-E) Summary of kinetic parameters for 0 or 20% PEG-3350 determined as in Figure 1C. (F-E) bar graphs depicting k_cat_ and K_m_ mean ± SD for TC45, TC35 and TC45^E/A^ at 0 (blue) or 20% (red) PEG-3350. (Ordinary ANOVA; *, < 0.05; **, < 0.01; ns= not significant).

**S6**: Representative fluorescence emission of TC45 (black) vs. TC45^E/A^ (green) fusion proteins in a kinetic buffer with no PEG (empty circles) or 22.5% PEG 3350 (solid circles). (A) CFP-TC45-YFP vs. CFP-TC45^E/A^-YFP (the same graph as reported in Fig. 3C, copied here to facilitate comparison with panels B-E) (B) YFP-TC45-CFP vs. YFP-TC45^E/A^-CFP. (C) Equimolar combination of YFP-TC45-CFP and YFP-TC45-CFP vs. CFP-TC45^E/A^-YFP and YFP-TC45^E/A^-CFP. (D) CFP-TC45-CFP vs. CFP-TC45^E/A^-CFP. (E) Equimolar combination of CFP-TC45-CFP and YFP-TC45-YFP vs. CFP-TC45^E/A^-CFP and YFP-TC45^E/A^-YFP.

__

**S7**: (A) Illustration of the gating strategy (see Figure 4) to measure the intramolecular interactions in NCI-H358 cells. Top row is used to verify expression of CFP and YFP and fusion proteins alone and in combination. Middle row is used to exclude excitation resulting from direct YFP excitation. Bottom row shows the gating to select the FRET-positive signal. (B) Illustration of the gating strategy (see Figure 4) to measure the intermolecular interactions in NCI-H358 cells.
